# Cytoreductive surgery for advanced stage ovarian cancer in the second trimester of pregnancy—a case report and literature review

**DOI:** 10.1097/MD.0000000000021127

**Published:** 2020-07-17

**Authors:** Nicolae Bacalbaşa, Irina Bălescu, Mihaela Vîlcu, Simona Dima, Laura Iliescu, Iulian Brezean

**Affiliations:** a “Carol Davila” University of Medicine and Pharmacy; b Department of Obstetrics and Gynecology, “Ion Cantacuzino” Clinical Hospital; c Department of Visceral Surgery, “Fundeni” Clinical Institute; d Department of Surgery, “Ponderas” Academic Hospital; e Department of Visceral Surgery, “I. Cantacuzino” Clinical Hospital; f Department of Internal Medicine, “Fundeni” Clinical Institute, Bucharest, Romania.

**Keywords:** advanced stage ovarian cancer, cytoreduction, pregnancy

## Abstract

**Rationale:**

Advanced stage ovarian cancer is rarely encountered in pregnant women, due to the high number of ultrasound imagistic studies performed during this period. The clinical course of patients diagnosed with advanced stage ovarian cancer is similar in pregnant and nonpregnant women.

**Patient concerns:**

We present the case of a 27-year-old woman initially submitted to emergency surgery for ovarian cyst torsion in the ninth week of gestation, at that moment ovarian cystectomy being performed.

**Diagnoses:**

The histopathological studies demonstrated the presence of a moderately differentiated epithelial ovarian cancer.

**Interventions:**

Although the interdisciplinary team decided for staging surgery followed by platinum-based chemotherapy beginning from the second trimester of pregnancy, both the patient and her family refused this strategy and opined for total hysterectomy en bloc with bilateral adnexectomy. Surprisingly, intraoperatively both ovaries had a tumoral aspect, whereas peritoneal carcinomatosis nodules were found in the Douglas pouch. Therefore, the neoplastic process was staged as a IIIC epithelial ovarian cancer, a total hysterectomy with bilateral adnexectomy, Douglas pouch peritonectomy, omentectomy, pelvic and para-aortic lymph node dissection being performed.

**Outcomes:**

The patient was discharged in the sixth postoperative day and was confined to the oncology service in order to be submitted to the standard taxanes and platinum based chemotherapy.

**Lessons:**

Although ovarian cancer has been rarely reported during pregnancy, this diagnostic should be taken in consideration whenever persistent adnexal masses are encountered.

## 1 Introduction

Ovarian tumors have been reported with an overall incidence <5% in pregnant women, 5% of these cases being further diagnosed with malignant ovarian lesions.^[1]^ Due to the wide usage of ultrasound to provide an efficient monitoring of the pregnancy, most cases are diagnosed in apparently early stages of the disease when pregnancy preservation is feasible. Due to the small number of cases diagnosed with ovarian malignant tumors during pregnancy, a standard guideline has been not established yet. However, whenever torsion, rupture, infarction, or malignant transformation is suspicioned surgery should be taken in consideration; although the risk of adnexal mass torsion during pregnancy has been reported with an estimated incidence of up to 15%, cyst rupture has been rarely described.^[2]^ In the present article, we present the case of a 27-year-old patient diagnosed with advanced stage ovarian cancer and submitted to surgery during pregnancy. The patient gave consent for the present study and its publication and this report was approved by the Ethics committee of the institutional review board of “Dr. I. Cantacuzino” hospital.

## 2 Case presentation

**Figure 1:**
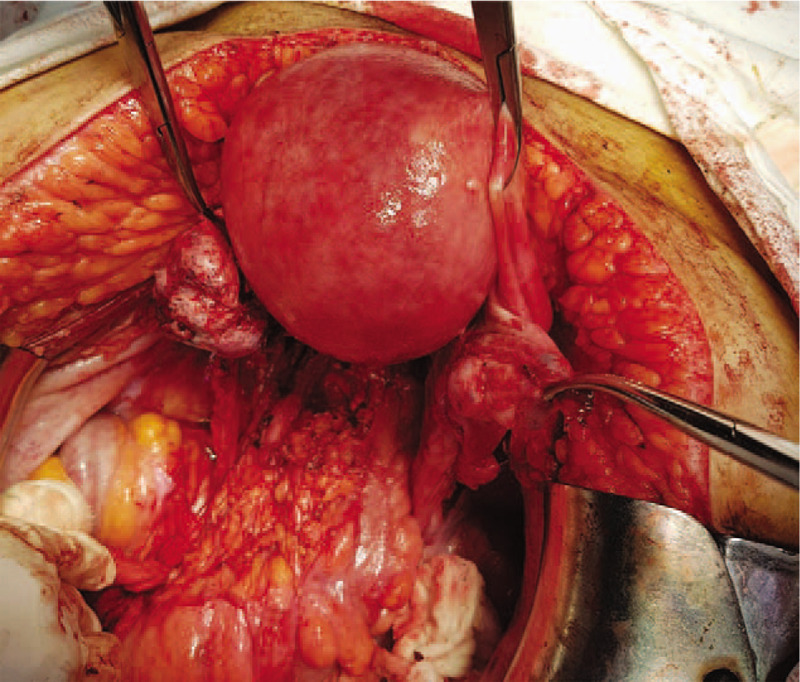
Initial intraoperative aspect—both ovaries presented tumoral transformation.

**Figure 2:**
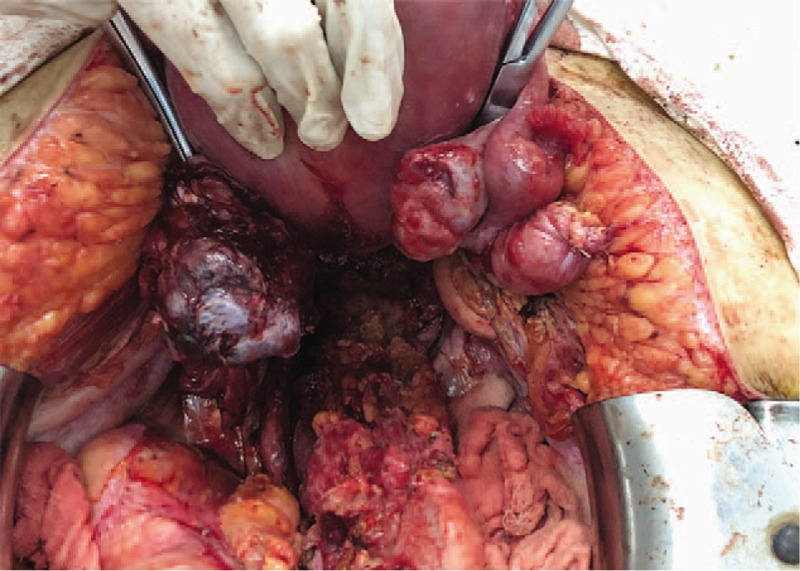
Peritoneal nodules involving the Douglas pouch and the rectal wall.

**Figure 3:**
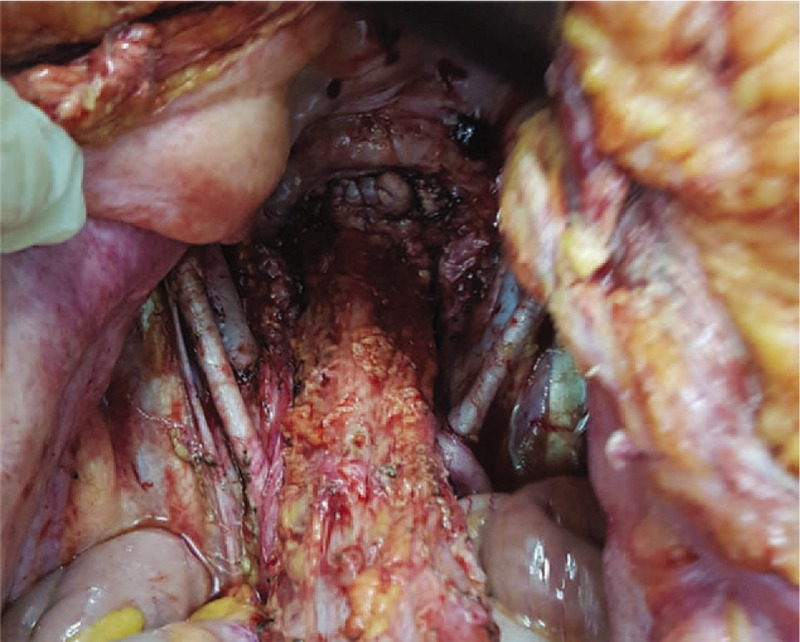
The final aspect after total hysterectomy en bloc with bilateral adnexectomy, peritonectomy, pelvic lymph node dissection.

**Figure 4:**
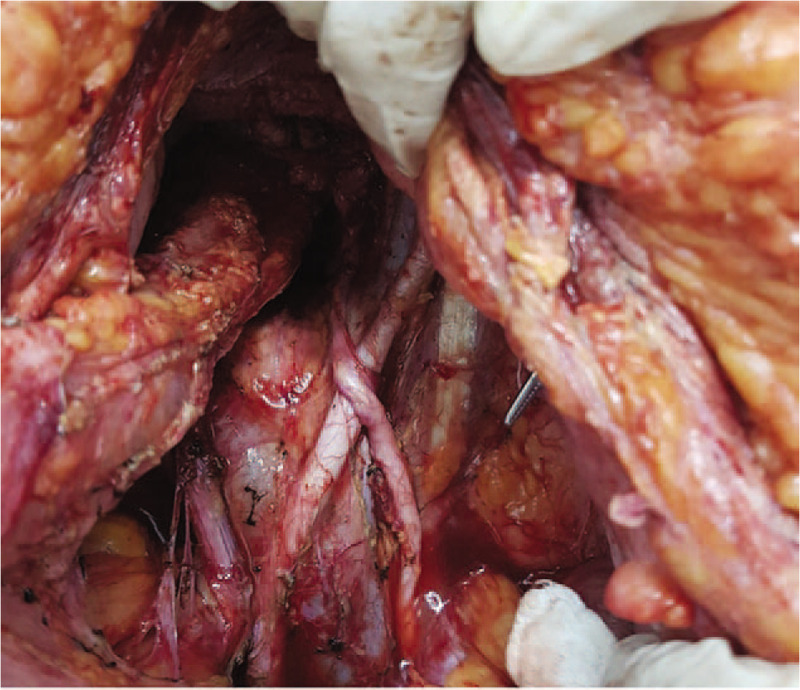
Completing the lymph node dissection in the para-aortic area.

A 27-year-old nuligesta patient was diagnosed during a routine ultrasound performed in the sixth week of gestation with a serous ovarian cyst measuring 4/3 cm located at the level of the right ovary, with no signs of local complications, so follow-up was proposed; however, 3 weeks later, she presented for diffuse pelvic pain, in association with local signs of peritoneal irritation. The vaginal ultrasound suspicioned torsion of the right adnexa, so emergency surgery was performed; intraoperatively, the diagnostic of torsion was confirmed; therefore, a laparoscopic cystectomy was performed, the remnant ovarian parenchyma presenting no pathological aspects. At that moment, during the laparoscopic exploration of the abdominal and pelvic cavity, no pathological aspect was revealed. The postoperative course was uneventful, the patient being discharged in the second postoperative day, after assuring for the maternal and fetal well-being. Three weeks later the histopathological studies revealed the presence of a moderately differentiated serous ovarian carcinoma. The patient was submitted to abdominal and pelvic ultrasound, and to a pelvic magnetic resonance; however, no signs of extended disease were found. A special medical committee formed by oncological gynecologist, surgeon, oncologist, obstetrician, anesthetist, and pediatrician was met to discuss which is the best therapeutic strategy in this particular case is; the final decision was to perform an exhaustive comprehensive surgical staging followed by platinum-based chemotherapy beginning from the second trimester of pregnancy and definitive surgery (consisting of total hysterectomy with bilateral adnexectomy, lymph node dissection, and omentectomy) after delivery. After discussing the benefits and the risks, neither the patient nor her family approved this decision and opined for giving up to the pregnancy and performing at this time a complete surgical procedure (consisting of total hysterectomy, total adnexectomy, pelvic and para-aortic lymph node dissection, omentectomy, and peritoneal biopsy). Therefore, to respect the patient's wish, surgery with curative intent was planned; surprisingly, during laparotomy, both ovaries presented signs of tumoral transformation, whereas tumoral nodules were found in the Douglas pouch (Figs. 1 and 2). In the meantime enlarged pelvic and para-aortic lymph nodes were seen, so total hysterectomy en bloc with bilateral adnexectomy, pelvic and parietal peritonectomy, resection of the peritoneal nodules from the Douglas pouch with rectal conservation, omentectomy, and complete pelvic and para-aortic lymph node dissection was performed (Figs. 3 and 4). The postoperative evolution was uneventful, the patient being discharged in the seventh postoperative day.

## 3 Discussion

Among pregnant women, a small proportion (ranging between 0.2% and 2% of cases) are at risk to develop adnexal masses, most commonly during the first trimester of gestation. However, most often these lesions are diagnosed during the routine examinations performed to follow-up the pregnancy and usually resolve spontaneously within the first 16 weeks of pregnancy; unfortunately, up to 6% of cases presenting adnexal masses during pregnancy prove to be malignant ovarian tumors, per cent which classifies ovarian cancer as the fifth most common tumor during pregnancy.^[3–5]^ When it comes to the most commonly encountered histopathological subtypes of ovarian malignancies encountered during pregnancy, it seems that they are similar to those reported in nonpregnant, reproductive age patients.^[6,7]^

As for the therapeutic management of a suspect ovarian mass during pregnancy, most gynecologists approve that >6 cm cysts, presenting a complex structure or which persist after the 16^th^ gestational week resection of the mass should be performed to prevent further complications and to exclude malignancy.^[8]^ Moreover, in cases presenting complicated lesions such as ruptured or torsioned cysts or highly malignant suspicion lesions, surgery might be proposed irrespective of the dimension of the lesion.^[9]^

When it comes to the most appropriate therapeutic option, a comprehensive surgical staging is mandatory to provide an adequate classification of the malignant process; whenever pregnancy preservation is desired, platinum-based chemotherapy from the second trimester of pregnancy followed by standard surgery after deliver can be taken into consideration.^[10–12]^ Studies conducted on pregnant patients submitted to chemotherapy demonstrated that paclitaxel and platinum salts can be safely administrated during the second and third trimester of pregnancy, without increasing the risk of fetal malformation.^[13]^

Comprehensive staging, defined by the Gynaecology Oncology Group as exploratory laparotomy, peritoneal washings, total hysterectomy en bloc with bilateral adnexectomy, omentectomy, pelvic and para-aortic lymph node dissection, and peritoneal biopsies, has been routinely proposed to maximize the detection of patients with microscopic metastatic disease, thereby increasing the number of cases who could benefit from adjuvant chemotherapy, decreasing in this way the risk of recurrence.^[14]^ Whenever an inadequate staging is performed, the risks of developing macroscopic relapse or to have a fulminant evolution of the disease is a maximal one; therefore, when reoperation is performed most cases will be diagnosed with disseminated lesions imposing performing multiple visceral resections to maximize the cytoreductive effort.^[15–17]^

An interesting study conducted on the theme of comprehensive surgical staging for apparently early-stage ovarian cancer was conducted by Garcia-Soto and published in 2012 in the *American Journal of Obstetrics and Gynaecology*.^[18]^ The study included 86 patients with ovarian malignant tumors grossly confined to the ovary submitted to comprehensive surgical staging between 1993 and 2009 in the University of Texas Southwestern Medical Center Hospital. The mean age of the patients at the time of comprehensive staging was 51 years, whereas the most commonly reported histopathological subtype was represented by endometroid tumors; although initially all lesions were apparently considered as early stage ovarian malignancies, after performing the comprehensive staging, 71% of cases proved to be indeed early stage ovarian cancer (stage I), 9% were classified as stage II tumors, 14% proved to be stage IIIA lesions, whereas the remaining 6% are upstaged to IIIC. Overall, 29% of cases were upstaged after comprehensive surgery, 60% of them being upstaged due to microscopic peritoneal/omentum/adhesions metastases, 20% of them being upstaged due to the presence of fallopian tube/uterine metastases, whereas the remaining 20% of cases were upstaged due to the presence of lymph node metastases. As for the main areas of positive peritoneal biopsies, most often microscopic disease was encountered at the level of the cul de sac as well as at the level of the adherences between the sigmoidian loop and the left ovary and on the left hemidiaphragm. Moreover, the authors demonstrated that the presence of poorly differentiated lesions, ascites and larger tumors were at risk to present more advanced stages of the disease; as for the influence of the preoperative levels of cancer antigen 125- CA125 - or of the positive cytology, no significant effect on the risk of upstaging could be demonstrated.^[18]^

In pregnant patients with apparently early-stage ovarian cancer who desire pregnancy preservation, comprehensive surgical staging might consist only in unilateral oophorectomy or unilateral adnexectomy in association with peritoneal washings and serial biopsies from the peritoneal surface, omentum, and, sometimes, lymph node sampling.^[9]^ However, in such cases frequent relapses have been reported in the postpartum period, due to the high capacity of recurrence of this malignancy; therefore, a close follow-up and, in certain cases, post-partum completion of the surgical procedure by performing a total hysterectomy with contralateral adnexectomy, omentectomy, and lymph node dissection have been proposed.

One of the largest studies conducted on the subject of clinical and pregnancy outcome in patients diagnosed with ovarian cancer during pregnancy was conducted by Kwon et al and was published in 2010.^[9]^ The study included 27 patients diagnosed with ovarian cancer during pregnancy between January 1996 and December 2006, with a mean age of 29.1 years (range 23–40 years); among these cases, 55.5% of patients were diagnosed with borderline ovarian tumors, 25.9% were diagnosed with epithelial ovarian malignant neoplasms, whereas the remaining 18.6% of cases were diagnosed with germ cell tumors. Among the 27 cases, pregnancy preservation surgery was feasible in 26 cases; as for the time of diagnostic, mean gestational age was 12.5 weeks (range 5–41 weeks). Surgery was performed during the first trimester in 11.1% of cases, during the second trimester in 70.1% of cases, and respectively, after delivery in 18.5% of cases. Among patients diagnosed with ovarian malignant lesions, the most commonly encountered subtype was the mucinous one (in 18.5% of cases) followed by the serous type and clear cell type (each subtype being reported in 3.7% of cases). As for the type of performed surgical procedure, among patients with epithelial ovarian cancer cystectomy was performed in 1 case, unilateral adnexectomy was performed in 5 cases, whereas cytoreductive surgery—including hysterectomy—was needed in 1 case. Cytoreductive surgery was performed in a patient with stage IIIC clear cell ovarian cancer. Among cases in which pregnancy preservation was feasible there was no fetomaternal complication after surgery; moreover, 3 patients received chemotherapy during pregnancy, whereas other 5 cases were submitted to chemotherapy just after delivery. As for the fetal follow-up, all 26 new born were healthy, full-term infants, 15 of them being delivered vaginally, whereas the remaining 11 cases were delivered by cesarean section; moreover, no fetal or placenta metastases were encountered.^[9]^

One of the most recently published review studies regarding the therapeutic perspectives for pregnant women diagnosed with ovarian malignancies was published by Boussios et al and was published in March 2018.^[19]^ The study identified 193 cases of ovarian cancer diagnosed during pregnancy and published until that moment in the English literature and underlined the utility of an adequate comprehensive staging; in cases with presumed early-stage ovarian cancer, multiple biopsies should be taken to exclude any disseminated lesions. Once the negativity of microscopic disease is confirmed, pregnancy preservation can be taken in consideration. Whenever disseminated lesions are encountered, the concept of maximal debulking surgery, similar to the one proposed in non-pregnant women, should be applied.^[19]^

In the case we reported, although initially an early-stage ovarian cancer was suspicioned and the medical committee's option was to perform a comprehensive surgical staging followed by chemotherapy during the second and third trimester of pregnancy, the patient refused this strategy and consented for per-primam radical surgery. Unfortunately, intraoperatively macroscopic disease was found so debulking surgery consisting of total hysterectomy, bilateral adnexectomy, pelvic and para-aortic lymph node dissection, pelvic and parietal peritonectomy (including the Douglas pouch), as well as total omentectomy was imposed.

## 4 Conclusion

Although the development of adnexal masses during pregnancy is a common situation, most often these lesions disappearing during the second trimester, in certain cases malignant ovarian cysts might develop. In such cases, surgery is mandatory to provide the right diagnostic; once malignancy is confirmed, comprehensive staging followed by chemotherapy might be proposed for early-stage ovarian cancer. In this way, pregnancy preservation can be offered to the patient. However, in the particular situation in which advanced stage disease is encountered, debulking principles should be applied in a similar manner to nonpregnant patients.

We used the CARE checklist when writing our report.^[20]^

## Author contributions

**Conceptualization:** Nicolae Bacalbasa, Iulian Brezean.

**Data curation:** Irina Balescu.

**Formal analysis:** Simona Dima, Laura Iliescu.

**Investigation:** Irina Balescu.

**Methodology:** Mihaela Vilcu.

**Writing – original draft:** Irina Balescu, Nicolae Bacalbasa.

**Writing – review & editing:** Iulian Brezean.
